# Case report: A case of esophageal small cell carcinoma misdiagnosed as leiomyoma

**DOI:** 10.3389/fmed.2024.1489207

**Published:** 2024-12-04

**Authors:** Junjun Yan, Xifeng Xiao

**Affiliations:** ^1^Department of Gastroenterology, Jiujiang City Key Laboratory of Cell Therapy, The First Hospital of Jiujiang City, Jiujiang, China; ^2^Department of Gastroenterology, Jiangxi Provincial Key Laboratory of Digestive Diseases, Jiangxi Clinical Research Center for Gastroenterology, Digestive Disease Hospital, The First Affiliated Hospital, Jiangxi Medical College, Nanchang University, Nanchang, China

**Keywords:** primary esophageal small cell carcinoma, submucosal tumor, early intervention, endoscopic submucosal dissection, case report

## Abstract

Primary esophageal small cell carcinoma (PESC) is a rare, extremely aggressive malignancy characterized by rapid growth, early metastasis, and poor prognosis. This study presents a case of early-stage PESC that was initially misdiagnosed as an esophageal leiomyoma, which was observed as a submucosal tumor during gastroscopy. The patient subsequently underwent endoscopic submucosal dissection (ESD), which successfully achieved complete tumor resection. Histopathological analysis later confirmed the diagnosis of small cell carcinoma. Subsequent treatments were recommended; however, the patient declined these options and developed systemic metastases 16 months later, indicating progression to advanced disease and a poor prognosis. This case underscores the imperative to consider PESC in the differential diagnosis of submucosal esophageal lesions, especially when clinical suspicion is elevated, despite its rarity. Additionally, it highlights the challenges associated with the diagnosis and management of submucosal PESC and emphasizes the crucial role of early diagnosis in enhancing patient prognosis and survival rates.

## Introduction

Primary esophageal small cell carcinoma (PESC) is a rare, aggressive neuroendocrine tumor with a high metastatic potential and poor prognosis. First described by McKeown et al. in 1952, it is distinct from esophageal squamous cell carcinoma and adenocarcinoma in both biological behavior and treatment approach (1, 2). Diagnosis is based on clinical presentations, endoscopic examination, and biopsy findings (3). PESC typically affects the middle or lower esophagus, often presenting as ulcerative or plaque-like lesions, though rare cases appear as submucosal tumors (3–6). This report describes a case of PESC initially misdiagnosed as leiomyoma, highlighting the importance of early and accurate diagnosis for better patient outcomes.

## Case report

A 65-year-old male patient with a chief complaint of retrosternal discomfort for the past 2 years was presented to our hospital after being diagnosed with a submucosal tumor (SMT) in the esophagus at another hospital. The patient had a medical history of type 2 diabetes for over 10 years. He denied any history of hypertension, coronary heart disease, viral hepatitis, tuberculosis, malignancy, or genetic diseases in his personal or family history. He was a non-smoker and did not consume alcohol habitually. The physical examination did not reveal any significant positive signs. Laboratory tests showed a mild elevation of carcinoembryonic antigen (CEA) at 12.9 μg/L, whereas blood routine test results and liver, kidney, and thyroid function tests were within normal limits.

**Figure 1:**
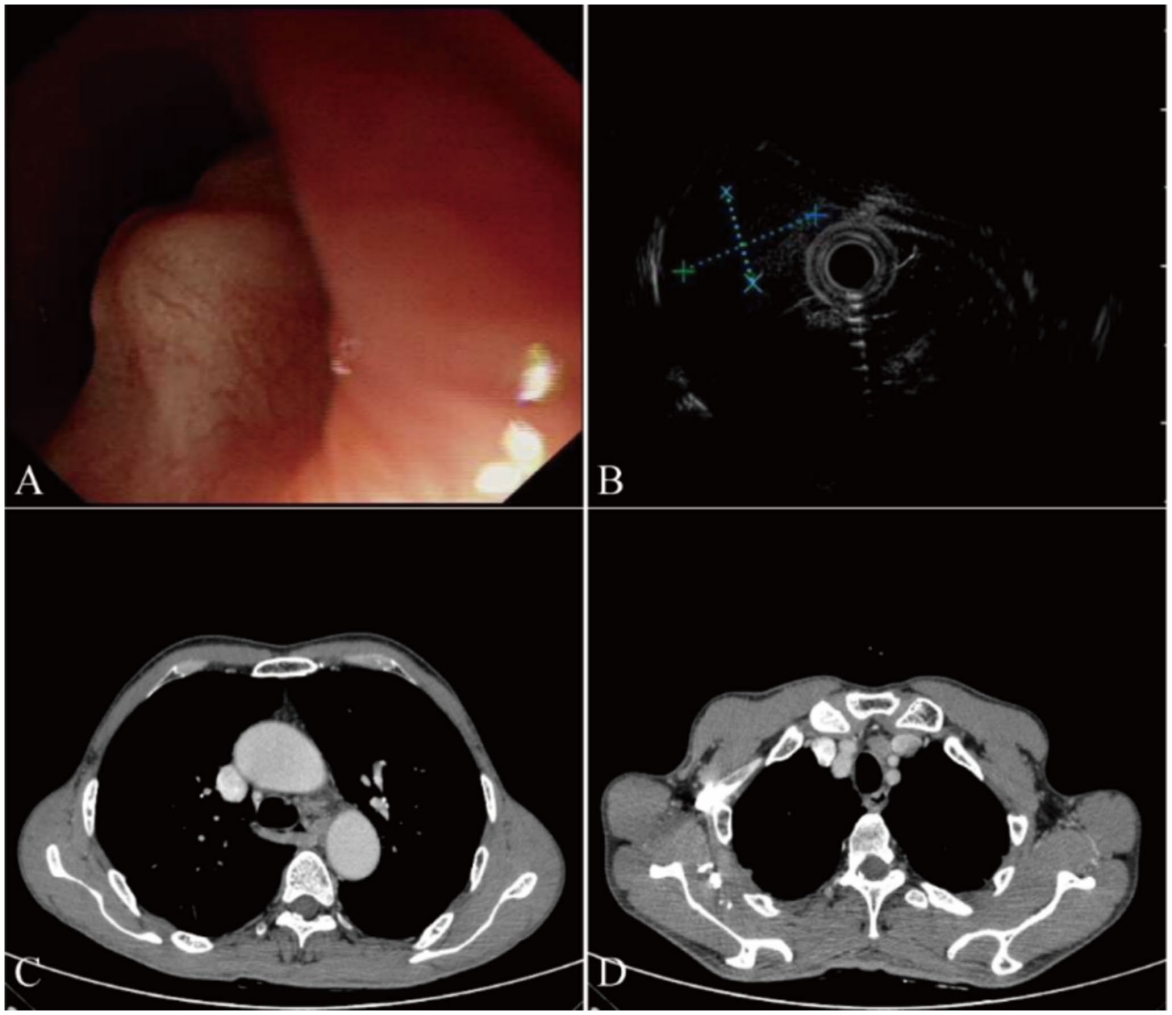
Endoscopic examination and computed tomography (CT) results in our case. **(A)** White light endoscopy shows a smooth subepithelial elevation measuring approximately 1.5 cm in diameter, located 27 cm from the incisors along the left esophageal wall. The lesion appears well-defined and does not cause significant luminal narrowing. **(B)** Endoscopic ultrasonography (20 MHz) reveals an ovoid hypoechoic mass originating from the submucosal layer. The tumor measures approximately 9.1 mm × 6.1 mm in size, with well-defined borders and no signs of infiltration into surrounding structures. **(C)** Enhanced chest CT scan demonstrates a slight thickening of the middle segment wall of the esophagus, suggesting the presence of a submucosal lesion. **(D)** Enlarged lymph nodes anterior to the main bronchus are visible on the CT scan, indicating possible regional lymphadenopathy.

Upon admission, endoscopy revealed the presence of a SMT with a central depression on the surface, measuring approximately 1.5 cm in diameter. The SMT was located 27 cm from the incisors along the left esophageal wall (Figure 1A). Endoscopic ultrasonography (20 MHz) characterized the lesion as an ovoid hypoechoic mass originating from the muscularis mucosae, displaying clear margins and homogeneous echogenicity (Figure 1B). Enhanced CT scan showed slight thickening of the middle segment wall of the esophagus and enlargement of lymph nodes anterior to the main bronchus (Figures 1C,D). Due to the submucosal nature and normal mucosa covering the lesion, we did not perform biopsy sampling during endoscopy procedure. Based on ultrasound findings showing a well-defined hypoechoic nodule, we considered benign leiomyoma more likely and thus recommended ESD treatment for this case.

**Figure 2:**
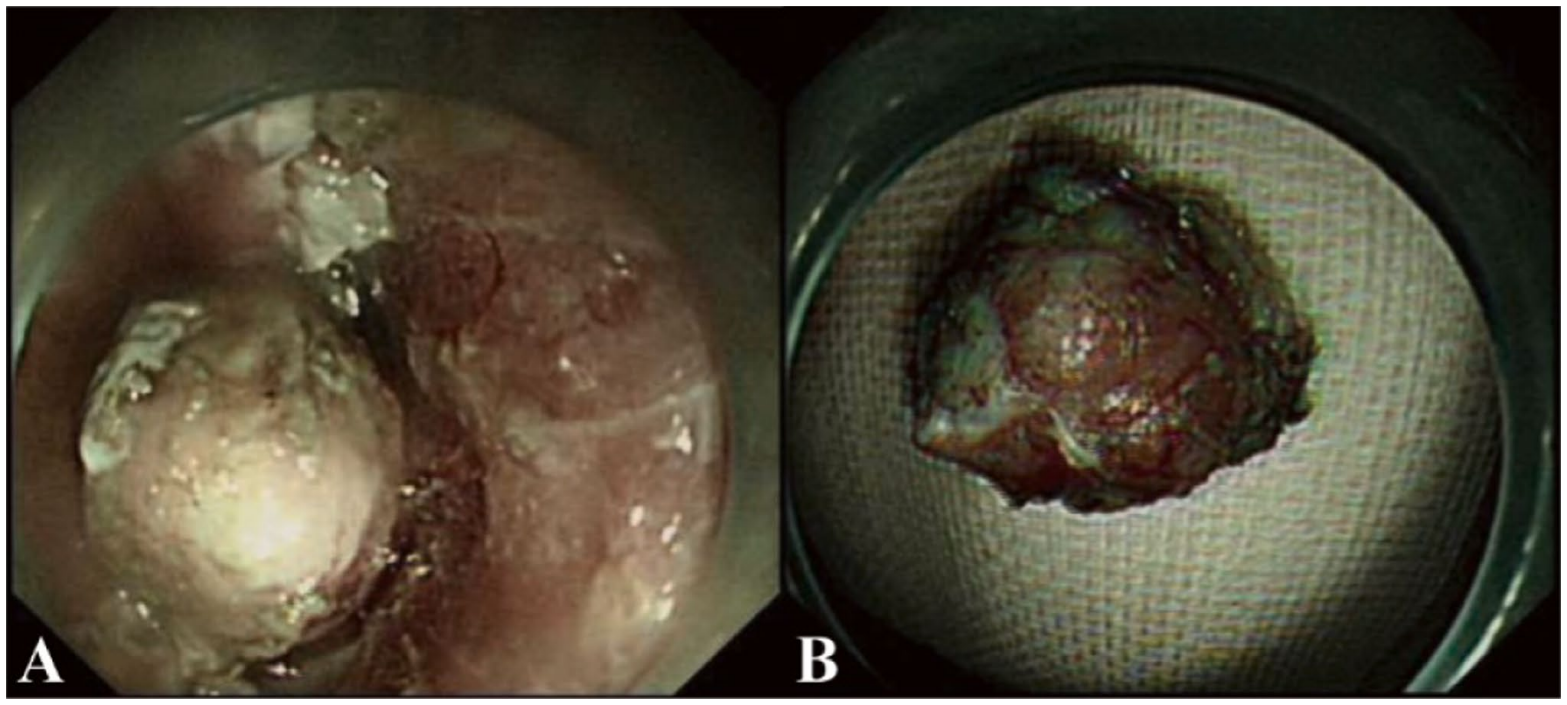
Endoscopic submucosal dissection (ESD) procedure in our case. **(A)** Stepwise dissection through the mucosa and submucosa layers was performed using a dual-knife endoscope. The procedure involved careful circumferential marking, submucosal injection, and sequential cutting until complete resection of the lesion was achieved. **(B)** Postoperatively, an intact white tumor body is revealed after successful resection. The specimen measures approximately 1.5 cm in diameter and appears well-preserved, suitable for histopathological examination.

**Figure 3:**
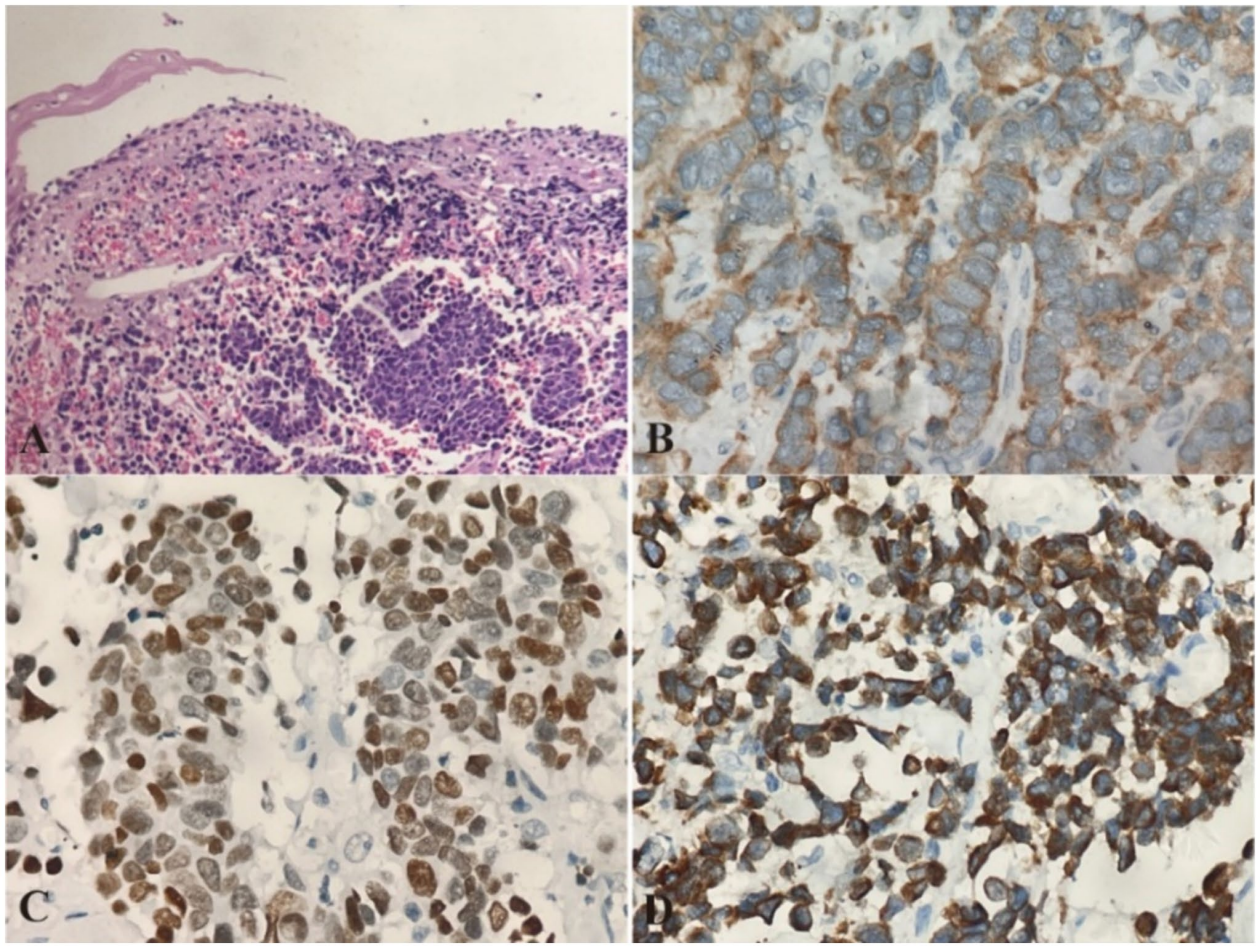
Pathological Images from esophageal primary small cell carcinoma (PESC). **(A)** Histopathological examination using hematoxylin and eosin (H&E) staining reveals the presence of small cell carcinoma cells located beneath the epithelial layer. The cells are densely packed, with scant cytoplasm and hyperchromatic nuclei, characteristic of small cell carcinoma. Magnification: 10×. **(B)** Immunohistochemical analysis shows strong positivity for synaptophysin, confirming the neuroendocrine nature of the tumor. Magnification: 20×. **(C)** Immunohistochemical staining for thyroid transcription factor-1 (TTF-1) is positive, supporting the diagnosis of a pulmonary-type neuroendocrine carcinoma. Magnification: 20×. **(D)** Pan-cytokeratin (CKpan) staining is also positive, indicating the presence of cytokeratin in the tumor cells. Magnification: 20×.

During ESD procedure, argon plasma coagulation (APC) marking was performed around the lesion, followed by submucosal injection using a glycerol-fructose-adrenaline mixture (Figure 2A). A negative lift sign was noted before precutting along the marked points, and then stepwise dissection through the mucosa and submucosa layers was carried out until complete resection was achieved, revealing an intact white tumor body postoperatively (Figure 2B). Histopathological examination revealed the presence of small cell carcinoma cells located beneath the epithelial layer. These cells are characterized by their small volume, low chromatin content, deep staining, high mitotic activity, and multifocal necrosis (Figure 3A). Immunohistochemical analysis revealed positivity for synaptophysin (Figure 3B), thyroid transcription factor-1 (TTF-1, Figure 3C), pan-cytokeratin (CKpan, Figure 3D), chromogranin A and CD56, with a Ki-67 index of 95%. Whole-body bone scan showed no evidence of bone metastasis. After multidisciplinary discussion, the diagnosis of primary esophageal small cell carcinoma was established, with the stage determined as II (T2N1M0). Given the high propensity of small cell carcinoma for early metastasis and poor prognosis, we recommended a comprehensive treatment approach involving surgical resection followed by adjuvant chemoradiotherapy. Unfortunately, the patient declined further treatment and was subsequently discharged. The patient developed systemic metastases after 16 months and required oral oxycodone for pain management, indicating advanced disease and a challenging prognosis.

## Discussion

Primary esophageal small cell carcinoma (PESC) is a rare, highly aggressive neuroendocrine tumor characterized by rapid growth, early metastasis, and poor prognosis (1, 3, 5, 7). Early diagnosis plays a crucial role in improving patient prognosis and survival rates. Treatment options for PESC are typically involve a combination of surgical resection, chemotherapy, biotherapy, and/or radiation therapy (8–10). The choice of treatment modality is tailored to the individual case, with a multidisciplinary approach being crucial for optimizing outcomes (3, 5). This case highlights the importance of considering PESC in the differential diagnosis of submucosal esophageal lesions, especially when clinical suspicion is high. The patient’s tumor, initially misdiagnosed as a leiomyoma, was confirmed as PESC through endoscopic submucosal dissection (ESD). Despite successful resection, the patient declined further treatment and developed systemic metastases 16 months later, indicating advanced disease and poor prognosis.

PESC typically presents as bulky masses or ulcers, often accompanied by symptoms such as retrosternal pain and dysphagia, which are typically indicative of advanced disease (3). Early PESC is often asymptomatic, which makes the early diagnosis of PESC particularly challenging in clinical practice. The manifestation of PESC as a submucosal tumor is an extremely rare occurrence, with only three cases reported in the literature (11, 12). In 2011, Naohiko Kawamura et al. reported a 54-year-old male with a submucosal tumor diagnosed as stage I (T2N0M0) PESC (12). In 2021, Er et al. reported two cases of PESC characterized by submucosal lesions (11). Despite its rarity, PESC should be considered in the differential diagnosis of submucosal esophageal lesions, especially when clinical suspicion is high.

This report describes an early-stage PESC initially misdiagnosed as a leiomyoma due to a hypoechoic mass seen on endoscopic ultrasonography. The smooth overlying mucosa and low biopsy positivity rate make routine biopsies less effective, increasing the risk of misdiagnosis. In our case, chest CT revealed mediastinal lymphadenopathy and elevated tumor markers, necessitating a more comprehensive diagnosis. Differential diagnosis must consider both benign and malignant conditions, particularly when lymph node abnormalities are present. Endoscopic ultrasound (EUS)-guided fine needle aspiration (FNA) can provide valuable diagnostic information. However, the patient declined EUS-FNA and opted for endoscopic submucosal dissection (ESD). Pathological examination confirmed undifferentiated small cell carcinoma, with cancer cells visible in the submucosal layer but not involving the epithelial layer. Due to economic constraints, the patient underwent a whole-body bone scan instead of PET/CT. After multidisciplinary consultation, the patient was diagnosed with primary limited-disease PESC (T2N1M0).

The role of surgical treatment in limited-disease PESC remains controversial. While some argue that hidden distant metastases may limit the benefit of surgery, emerging evidence suggests that surgical resection can provide a survival advantage in localized disease (11, 12). In our case, despite early diagnosis through ESD, the patient’s refusal of further treatment led to rapid disease progression. A multidisciplinary approach, including surgical resection and adjuvant therapy, is essential for optimal outcomes.

There are some limitations in our report. For submucosal tumors suspected to originate from the superficial muscularis mucosa, obtaining deeper tissue samples through targeted biopsies is crucial for accurate diagnosis. Additionally, if a benign lesion is suspected but CT scans show mediastinal lymph node involvement, further investigation, including targeted lymph node biopsies and supplementary imaging such as PET-CT, is necessary to distinguish between benign and malignant processes. Implementing these measures would have facilitated a more definitive diagnosis and enabled a more effective treatment strategy.

## Data Availability

The original contributions presented in the study are included in the article/supplementary material, further inquiries can be directed to the corresponding author.
